# First dengue virus seroprevalence study on Madeira Island after the 2012 outbreak indicates unreported dengue circulation

**DOI:** 10.1186/s13071-019-3357-3

**Published:** 2019-03-13

**Authors:** Heidi Auerswald, Ana de Jesus, Gonçalo Seixas, Teresa Nazareth, Saraden In, Sokthearom Mao, Veasna Duong, Ana Clara Silva, Richard Paul, Philippe Dussart, Carla Alexandra Sousa

**Affiliations:** 1 Virology Unit, Institut Pasteur du Cambodge, Institut Pasteur International Network, PO Box 983, Phnom Penh, Cambodia; 2 GHTM-Global Health and Tropical Medicine, 1349-008 Lisbon, Portugal; 3 UEI Medical Parasitology, Institute of Hygiene and Tropical Medicine of Lisbon, Universidade Nova de Lisboa, Lisbon, Portugal; 4 Departamento de Saúde, Planeamento e Administração Geral, Instituto de Administração da Saúde e Assuntos Sociais, IP-RAM, Funchal, Madeira Portugal; 5 Madeira Regional Government, Institute of Health and Social Affairs, Av. Zarco, Funchal, Madeira Portugal; 6 Functional Genetics of Infectious Diseases Unit, Department of Genomes and Genetics, Institut Pasteur, 75015 Paris, France; 7 Génomique évolutive, modélisation et santé UMR 2000, Centre National de la Recherche Scientifique (CNRS), 75724 Paris Cedex 15, France

**Keywords:** Dengue virus, Seroprevalence, Madeira Island, Serotype

## Abstract

**Background:**

In 2012, the first dengue virus outbreak was reported on the Portuguese island of Madeira with 1080 confirmed cases. Dengue virus of serotype 1 (DENV-1), probably imported from Venezuela, caused this outbreak with autochthonous transmission by invasive *Aedes aegypti* mosquitoes.

**Results:**

We investigated the seroprevalence among the population on Madeira Island four years after the outbreak. Study participants (*n* = 358), representative of the island population regarding their age and gender, were enrolled in 2012 in a cross-sectional study. Dengue antibodies were detected with an in-house enzyme-linked immunosorbent assay (ELISA) using the dimer of domain III (ED3) of the DENV-1 envelope protein as well as commercial Panbio indirect and capture IgG ELISAs. Positive ELISA results were validated with a neutralization test. The overall seroprevalence was found to be 7.8% (28/358) with the in-house ELISA, whereas the commercial DENV indirect ELISA detected IgG antibodies in 8.9% of the individuals (32/358). The results of the foci reduction neutralization test confirmed DENV-1 imported from South America as the causative agent of the 2012 epidemic. Additionally, we found a higher seroprevalence in study participants with an age above 60 years old and probable secondary DENV infected individuals indicating unreported dengue circulation before or after 2012 on Madeira Island.

**Conclusions:**

This study revealed that the number of infections might have been much higher than estimated from only confirmed cases in 2012/2013. These mainly DENV-1 immune individuals are not protected from a secondary DENV infection and the majority of the population of Madeira Island is still naïve for DENV. Surveillance of mosquitoes and arboviruses should be continued on Madeira Island as well as in other European areas where invasive vector mosquitoes are present.

**Electronic supplementary material:**

The online version of this article (10.1186/s13071-019-3357-3) contains supplementary material, which is available to authorized users.

## Background

Dengue fever is the most widespread mosquito-borne viral disease causing an annual estimated 390 million infections [1] and 25,000 deaths [2]. Infection by any one of four antigenically distinct serotypes of dengue virus (DENV) can lead to symptoms including high fever or more severe disease with haemorrhage and plasma leakage. However, the majority (~80%) of infections results in a mild or subclinical outcome [3].

The virus is mainly transmitted by the mosquito species *Aedes aegypti*, inhabiting the tropics and subtropics, but can also be transmitted by *Ae. albopictus*, which is an invasive species present in several European countries [4, 5]. Due to the increasing number of imported DENV infections [6, 7], there is a growing likelihood of autochthonous infections where competent vectors are present. Such autochthonous transmission of DENV in regions with a naïve population has occurred intermittently in France [8–10], Croatia [11, 12] and the USA [13, 14] over the past decade. Furthermore, Japan recently experienced an unprecedented epidemic in Tokyo [15, 16]. However, Europe has not witnessed a large dengue outbreak since the epidemic in Greece in 1927/28 [17].

In September 2012, a unique European dengue outbreak occurred on Madeira Island, the larger of the two inhabited islands of the Portuguese autonomous region in the Atlantic Ocean. Madeira has been classified as having a Mediterranean climate, meaning it has mild year-round temperatures; the average annual temperature at the weather station of the capital city of Funchal was 19.6 °C for the period 1980–2010. On the highest windward slopes of Madeira, rainfall exceeds 1250 mm per year, mostly falling between October and April (according to the Meteorology Institute of Portugal, IPMA). The dengue epidemic resulted in 2168 probable cases and 1080 confirmed infections from September 2012 to March 2013, affecting mainly residents from Funchal and the neighbouring provinces, Câmara de Lobos, Sao Martinho and Caniço [18]. The majority of the ≈270,000 inhabitants of this main island live on the south coast around the capital city of Funchal. In contrast to the small autochthonous dengue outbreaks on the European mainland, the outbreak in Madeira was spread by *Ae. aegypti* mosquitoes, which were first reported to be present on the island in 2004 [19]. As this was the first dengue epidemic that hit the Portuguese island, the population of Madeira was naïve for DENV infections. The DENV-1 serotype imported from Venezuela in South America and belonging to genotype V was believed to have been responsible for the outbreak [20, 21].

This study was conducted to investigate the seroprevalence among the population on Madeira Island and thus the actual extent of the dengue epidemic. We aimed to verify DENV-1 as the causative agent of the epidemic by performing foci reduction neutralization tests (FRNT) against all four DENV serotypes, and additionally against a DENV-1 genotype V isolate originally isolated in 2009 in French Guiana [22]. A secondary objective was the evaluation of an in-house enzyme-linked immuno-sorbent assay (ELISA) against a commercial indirect ELISA and the neutralization test, the gold standard for serological flavivirus diagnostics (Additional file 1: STROBE Statement).

## Methods

### Study design and sampling

Serum samples from inhabitants of Madeira Island were collected in 2016. The minimum number of participants was estimated to be representative of the Madeira population in 2012 (258,686 inhabitants) regarding all ages and both genders. The total number of participants for this seroprevalence study for the 235,233 people above the age of 10 years-old (study age range: 11–92 years), stratified into gender and decennial age group, was calculated as previously described, applying the formula from Luiz & Magnanini [23]. Given that there were 1080 DENV confirmed cases during the 2012/2013 outbreak [18] and based on the assumption that only 20% infections led to symptomatic dengue fever cases, an estimated 5400 people might actually have had dengue during the epidemic and the probable prevalence rate of viral infection would be 2.1%. Therefore, the minimum sample size was calculated to be 237 participants (197 + 20% reserves), following the age/gender distribution shown in Additional file 2: Table S1.

The participants were recruited by convenience sampling at the Henriques de Gouveia laboratory and the Madeira Medical Centre among patients coming for routine blood analyses in November in 2015. After presenting the purpose of our study, each volunteer who agreed to participate filled out a questionnaire with personal data and relevant health information: age, gender, weight, county of residence, history of travel to dengue endemic countries and history of yellow fever and Japanese encephalitis vaccination. Blood samples were collected from each participant using BD Vacutainer tubes, and one extra-tube using a BD Vacutainer SST II Advance 8.5 ml was collected for the purpose of the study. The total blood volume was collected according to body weight of the participant as previously described [24]. On the day of collection, the blood samples dedicated to the study were centrifuged and an aliquot of 300 µl of serum of each sample was taken. All the samples were frozen and stored at -20 °C until laboratory analyses.

### Enzyme-linked immunosorbent assays

Both of the commercial Panbio ELISAs (Alere Inc., Waltham, MA, USA), Dengue IgG indirect ELISA and Dengue IgG capture ELISA, were performed according to the manufacturer’s instructions [25, 26]. The Panbio Dengue IgG indirect ELISA is used for the qualitative detection of IgG antibodies to DENV antigens of all four serotypes, whereas the Panbio Dengue IgG capture ELISA is used specifically for the qualitative detection of IgG antibodies in secondary DENV infections [25]. The latter is achieved by a higher cut-off value for positive results [27]. This testing strategy of using both commercial Panbio ELISAs allowed not only the detection of previous DENV infection within the population of Madeira Island, but also the exploration of possible unreported secondary DENV infections. The in-house ELISA test is an indirect assay developed at Institut Pasteur and uses the stabilized dimer of domain III (ED3) of the DENV-1 envelope protein for the detection of IgG antibodies against DENV in human serum [28].

The ELISA was performed in 96-well plates (Greiner Bio One, Kremsmünster, Austria) with a 100 µl/well processing volume. The plates were coated overnight at 4 °C with 100 µl of recombinant antigen diluted to 0.3 µg/ml in PBS (Sigma-Aldrich, Steinheim, Germany). The next day the coated plates were washed with PBS + 0.1% Tween 20 (PBS-T) and then incubated for 1 h at 37 °C with 100 µl/well of blocking buffer (PBS-T + 5% low-fat dried milk). Subsequently, the plates were washed with PBS-T and 100 µl serum samples diluted 1:100 in blocking buffer were added to wells in duplicate. The plates were incubated for 1 h at 37 °C. Next, serum dilutions were discarded, and plates were washed four times with PBS-T. Then, 100 µl of goat horseradish peroxidase-conjugated anti-human IgG antibody (Sigma-Aldrich) diluted 1:1000 in blocking buffer was added to each well and incubated for 1 h at 37 °C. Afterwards, plates were washed four times with PBS-T and 100 µl TMB substrate (Sigma-Aldrich) was added to each well and left for 10–15 min. The colour reaction was stopped by adding 100 µl/well of 0.5 N sulfuric acid and the optical density was measured at 450 nm with an Infinite 200 PRO NanoQuant spectrophotometer (Tecan, Männedorf, Switzerland).

### Cell lines for viral growth and neutralization studies

The monkey cell line LLC-MK2 was used for the detection of neutralizing antibodies via a foci reduction neutralization test [29]. Cells were cultivated in Dulbecco’s modified Eagle medium (DMEM; Sigma-Aldrich) supplemented with 10% fetal bovine serum (FBS; Gibco, Gaithersburg, MD, USA) and 100 U/ml penicillin-streptomycin (Gibco) at 37 °C and in a 5% CO_2_ atmosphere. All viruses were grown in *Ae. albopictus* C6/36 cells and harvested from the supernatant. These mosquito cells were cultured in Leibovitz 15 medium (Sigma-Aldrich) supplemented with 10% FBS, 1% L glutamine (Gibco), 10% tryptose-phosphate (Gibco) and 100 U/ml penicillin-streptomycin at 28 °C.

### Viruses

The reference viruses used for the neutralization test were the following: DENV-1 Hawaii (GenBank: AF425619), DENV-2 New Guinea C (GenBank: AF038403), DENV-3 strain H87 (GenBank: M93130), DENV-4 H241 (GenBank: AY947539) and JEV strain Nakayama (GenBank: EF571853). As no DENV-1 isolate from the Madeira epidemic was available, we used a virus that was isolated from a patient in French Guiana in 2009 (FGU 2009; GenBank: MH279620) that belongs to genotype V [22] (Additional file 3: Figure S1). The recombinant DENV-1 ED3 protein was produced based on the DENV-1 strain FGA/89 (accession no. AF226687) [28].

### Foci reduction neutralization test

The FRNT micro-neutralization assay, used as the gold standard, determined the level of neutralizing antibodies against different viruses. A subset of sera was tested by FRNT including (i) all sera formerly tested positive with at least one of the above mentioned ELISAs (*n* = 32); (ii) sera with an undetermined ELISA result (*n* = 3); and (iii) sera with negative results in all ELISAs randomly selected and representing 10% of the total study population, with similar numbers of sera for each age group (*n* = 36). The serum samples were analysed by FRNT as previously described [30], but modified by using LLC-MK2 cells and virus-specific polyclonal mouse hyperimmune ascites fluids (Institut Pasteur in Cambodia). Neutralization was defined as the serum dilution that induced a 90% reduction in the number of virus-induced foci (foci reduction neutralization test 90%; FRNT_90_) compared to controls (virus alone and flavivirus-negative control serum alone) and was calculated via log probit regression analysis (SPSS for Windows v.16.0; SPSS Inc., Chicago, USA). The first serum dilution used for the assay was 1:10 which resulted in a final in-test dilution of 1:20 after adding an equivalent volume of virus. Subsequently, the lower limit of quantification for the FRNT_90_ titer was defined as 20, which follows the WHO recommendations for flavivirus FRNTs [31]. The DENV that induced at least a 4-fold higher titer compared to the other DENVs determined the DENV serotype responsible for the dengue infection.

### Statistical analysis

Risk factor analysis of the association of age (continuous), gender, yellow fever vaccination (yes/no), travel history outside of Madeira (yes/no) and geographical site (11 districts) on dengue seropositivity using the results from the indirect Panbio IgG was performed by fitting a generalized linear model with binomial error structure (i.e. logistic regression) using GenStat 15th Edition, (VSN International Ltd., Hemel Hempstead, UK). Wald test in the context of this logistic regression was used to determine whether a certain predictor variable was significant or not. Wald statistics, which approximate to a Chi-square distribution, are given. A dispersion parameter was estimated to account for over-dispersion in the data. There were only two individuals with JEV vaccination and therefore this variable was not included in the analysis. Mean FRNT_90_ titers were compared using the 2-tailed Mann-Whitney test to determine if differences were significant. The agreement of the FRNT results with the results of the diverse ELISAs was performed via Cohen’s kappa test. All statistical analyses were performed with a significance level of α = 0.05.

An estimated number of total infections was calculated by multiplying the number of people of a determined age group (Additional file 2: Table S1) by the proportion of seropositive results of that age group in the test population. The process was repeated for all the pre-determined age groups and summed to give the estimated number of infections. For example, if we had 15,000 people aged from 20–29 on Madeira Island at the time, and we had collected 100 blood samples from people of that age group of which 15 had a positive result, then we estimate that 2250 people of that age group have been infected by dengue. This calculation was carried out for every studied age group and then summed to generate an overall estimated number of infections.

## Results

### Study population

The study enrolled 358 individuals aged from 11 to 92 (mean age: 49) and a male:female ratio of 0.61 (Table 1). The study included residents from all 11 districts of Madeira although the majority of the study participants resided in Funchal (229/358, 64.0%). In total, 8.7% of the study participants (31/358) self-reported a former vaccination against the yellow fever virus (YFV), and 31.8% (114/358) had travelled to DENV endemic countries. Only two participants were previously vaccinated against Japanese encephalitis virus (JEV).

**Table 1:** Demographic characteristics of study participants

Participants	DENV seropositive^a^ *n* (%)	DENV seronegative *n* (%)	Total *n* (%)
Total number	28 (7.8)	330 (92.2)	358 (100)
Male	11 (8.1)	125 (91.9)	136 (38.0)
Female	17 (7.7)	205 (92.3)	222 (62.0)
Median age (years)	57.5	49	49
10–19	0 (0)	19 (100)	19 (5.3)
20–29	3 (7.9)	35 (92.1)	38 (10.6)
30–39	3 (4.8)	59 (95.2)	62 (17.3)
40–49	6 (9.8)	55 (90.2)	61 (17.0)
50–59	3 (4.2)	68 (95.8)	71 (19.9)
60+	13 (12.1)	94 (87.9)	107 (29.9)
Travel history^b^	9 (7.9)	105 (92.1)	114 (31.8)
No travel history^b^	19 (7.8)	225 (92.2)	244 (68.2)
YFV vaccination	3 (9.7)	28 (90.3)	31 (8.7)
No YFV vaccination	25 (7.6)	302 (92.4)	327 (91.3)
Funchal	20 (8.7)	209 (91.3)	229 (64.0)
Câmara de Lobos	1 (4.5)	21 (95.5)	22 (6.1)
Santa Cruz	3 (6.1)	46 (93.9)	49 (13.7)
Sao Vicente	1 (16.7)	5 (83.3)	6 (1.7)
Porto Moniz	1 (50)	1 (50)	2 (0.6)
Machico	0 (0)	8 (100)	8 (2.2)
Ribeira Brava	0 (0)	8 (100)	8 (2.2)
Calheta	0 (0)	6 (100)	6 (1.7)
Santana	0 (0)	4 (100)	4 (1.1)
Ponta do Sol	0 (0)	4 (100)	4 (1.1)
Porto Santo	0 (0)	2 (100)	2 (0.6)
Missing data	2 (11.1)	16 (88.8)	18 (5.0)

^a^Dengue seropositive patient identified by at least one ELISA assay and confirmed with seroneutralization assay (FRNT)

^b^To dengue endemic countries

*Abbreviations*: DENV, dengue virus; YFV, yellow fever virus

### Seroprevalence

The dengue seroprevalence of the study participants was tested by in-house DENV IgG indirect ELISA using dimer of Domain III (ED3) of the DENV-1 envelope protein and the DENV IgG indirect ELISA from Panbio. In total, the in-house ELISA detected 7.8% (28/358) of the study participants as positive, whereas the commercial DENV indirect ELISA detected IgG antibodies in 8.9% of the individuals (32/358; Table 2). For three samples the result of the indirect Panbio ELISA was undetermined. A subset of 71 sera was tested by FRNT including all sera with a positive (*n* = 36) or undetermined (*n* = 3) result in any of the two indirect ELISAs (Additional file 4: Table S2) and 32 sera with negative results in all ELISAs. Individuals were considered positive for DENV antibodies if they showed a FRNT_90_ titer ≥ 20 for one or more DENV reference strains. Based on these criteria, 28 study participants were found positive for DENV neutralizing antibodies (Table 2).

**Table 2:** Comparison of serology results obtained with the different enzyme-linked immuno-sorbent assays used (*n* = 358) and the foci reduction neutralization test (*n* = 71)

Serological assays used	Positive samples *n* (%)	Negative samples *n* (%)	Undetermined *n* (%)
*n *= 358			
DENV-1 ED3 dimer ELISA (in-house)	28 (7.8)	330 (92.2)	–
DENV IgG indirect ELISA (Panbio)	32 (8.9)	323 (90.3)	3 (0.8)
DENV IgG capture ELISA (Panbio)	5 (1.4)	349 (97.5)	4 (1.1)
*n* = 71^a^			
DENV-1 ED3 dimer ELISA (in-house)	28 (39.4)	43 (60.6)	–
DENV IgG indirect ELISA (Panbio)	32 (45.1)	36 (50.7)	3 (4.2)
DENV IgG capture ELISA (Panbio)	5 (7.1)	62 (87.3)	4 (5.6)
FRNT^b^	28 (39.4)	43 (60.6)	–
DENV-1^c^	24 (33.8)	47 (66.2)	–
DENV-2^c^	5 (7.0)	66 (93.0)	–
DENV-3^c^	5 (7.0)	66 (93.0)	–
DENV-4^c^	7 (9.9)	64 (90.1)	–
DENV-1 genotype V	27 (38.0)	44 (62.0)	–

^a^Subset of additional samples tested by FRNT

^b^FRNT_90_ titer ≥ 20

^c^Dengue reference strain

*Abbreviations*: DENV, dengue virus; ELISA, enzyme-linked immuno-sorbent assay; FRNT, foci reduction neutralization test

Among the DENV seropositive samples, the DENV capture IgG ELISA from Panbio designed to identify secondary dengue infection detected five positive individuals (5/28, 17.9%). Additionally, four people (4/28, 14.3%) showed inconclusive results and therefore the dengue immune status could not be determined with this assay. Based on these results, most of the seropositive study participants (19/28, 67.8%) were considered to have had a primary DENV infection.

**Fig. 1:**
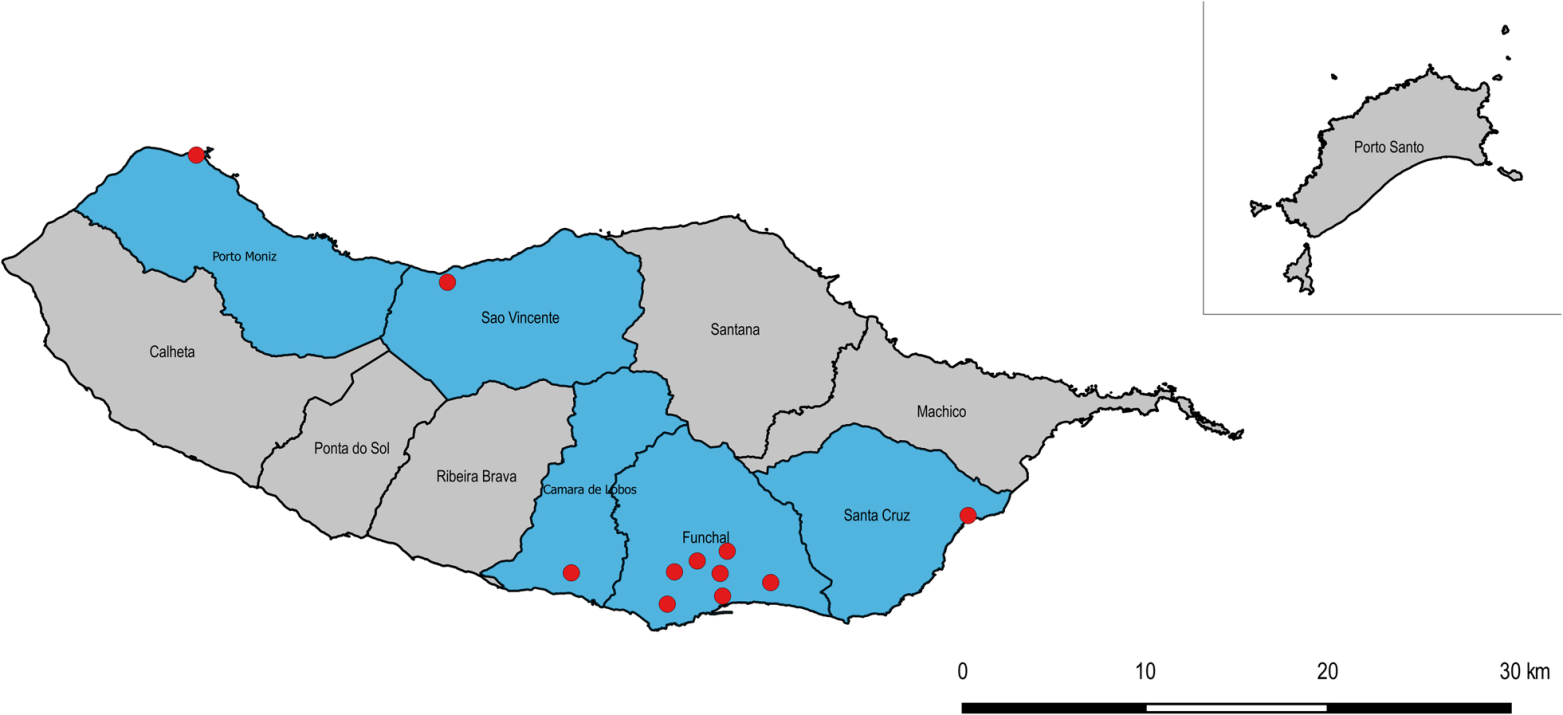
Geographical distribution of the seroprevalence across Madeira Island. Districts with dengue seropositive study participants are shown in blue and the respective residences are marked with red dots. The map was created using QGIS 2.14.3 and the base layer data were obtained from DIVA GIS (http://www.diva-gis.org/gdata)

Seropositive individuals were found in only five of the eleven districts of Madeira, largely reflecting sampling effort. Most of the seropositive individuals (20/28, 71.4%) were residents of Funchal, with ever decreasing numbers in the other Southern districts (Ponta do Sol, Ribeira Brava, Câmara de Lobos and Santa Cruz) (Fig. 1). We detected only two cases from the north, one from Porto Moniz and one from Sao Vicente (Table 1).

Risk factor analysis of the indirect Panbio ELISA seropositivity revealed no significant association with gender (Wald test: *χ*^2^ = 0.28, *df* = 1, *P* = 0.595), yellow fever vaccination (Wald test: *χ*^2^ = 0.72, *df* = 1, *P* = 0.397), travel history to DENV endemic countries (Wald test: *χ*^2^ = 0.01, *df* = 1, *P* = 0.94) or place of residence (Wald test: *χ*^2^ = 6.69, *df* = 10, *P* = 0.753). However, there was a significantly increased risk with increasing age (Wald test: *χ*^2^ = 12.57, *df* = 1, *P* < 0.001; odds ratio per year of age 1.03; 95% CI: 1.01–1.05).

The seroprevalence results were extrapolated to obtain the estimated number of infections during the dengue outbreak. Based on the population in 2012 (total number of residents: 258,686) and the FRNT confirmed seroprevalence of 7.8%, the total number of infections was estimated to be 16,606 (95% binomial CI: 9221–41,303). The 60+ years old group contributed an estimated > 6000 cases due to the high seroprevalence observed (12.1%; 13 seropositive out of 107 study participants with an age of 60+).

### FRNT with DENV reference strains and DENV-1 genotype V strain

A subset of 71 sera was tested by FRNT using all four dengue serotypes to measure the levels of neutralizing antibodies (FRNT_90_ titer; Table 2) as well as the serotype. The DENV serotype was identified as the virus which induced the highest FRNT_90_ titer. Overall, the FRNT using DENV reference strains detected 28 individuals with neutralizing antibodies; all these individuals formerly tested positive with at least one of the three describes ELISAs. None of the ELISA negative samples was detected positive with the FRNT. Among the 28 FRNT positive samples, 24 (85.7%) were detected positive for neutralizing antibodies against DENV-1 reference strain. Among these, we identified 21 individuals with DENV-1 serotype (indicated by the highest FRNT_90_ titer against DENV-1 reference strain compared to the other DENVs). Moreover, three study participants were identified with neutralizing antibodies against DENV-2 (*n* = 1), DENV-3 (*n* = 1) or DENV-4 (*n* = 1).

**Fig. 2:**
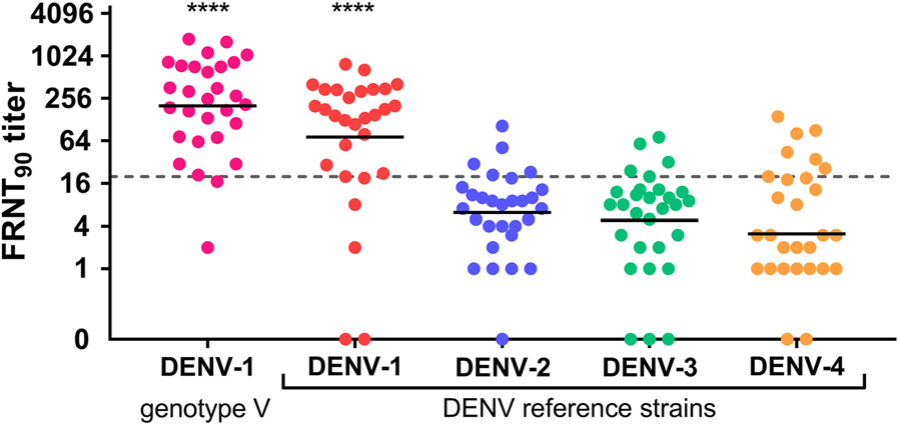
Individual FRNT_90_ titers of all FRNT positive study participants (*n* = 29). Mean FRNT_90_ titers with standard error for FRNT positive samples. Overall, 28 samples were positive in the FRNT using the reference strains of DENV-1 (red), DENV-2 (blue), DENV-3 (green) and DENV-4 (yellow). An additional FRNT with DENV-1 genotype V (pink) added one more seropositive study participant. The dashed line indicates threshold of 20. Asterisks indicate the statistically significant different mean FRNT_90_ titers between the DENV-1 reference strain and the DENV-1 genotype V strain with the other DENV serotypes (Mann-Whitney test, *P* < 0.0001)

After using the reference strains for all four DENVs (DENV 1-4) we investigated further the immune response to DENV-1 genotype V strain, as this was the proposed cause of the 2012 outbreak in Madeira Island. Among the 71 sera tested by FRNT with a DENV-1 genotype V strain, we identified one study participant with a low FRNT_90_ titer of 30 against this DENV-1 genotype V strain that was formerly negative against all four DENV reference strains (Additional file 4: Table S2). This increased the total number of FRNT positive study participants to 29 (Table 3, Fig. 2). Two other individuals were negative for antibodies against the DENV-1 reference strain but showed detectable levels of neutralizing antibodies against the DENV-1 genotype V strain (FRNT_90_ titer of 73 and 71, respectively). One of these also had neutralizing antibodies against DENV-3 (FRNT_90_ = 58); the other individual was also positive for both DENV-3 (FRNT_90_ = 24) and DENV-4 (FRNT_90_ = 81).

**Table 3:** Mean neutralization titers for seropositive samples by foci reduction neutralization test (*n* = 29)

Virus used	Positive samples with FRNT	
	Mean FRNT_90_	95% CI
DENV-1 reference strain	201	127–275
DENV-1 genotype V strain	444	262–626
DENV-2 reference strain	13	5.5–21
DENV-3 reference strain	12	6–18
DENV-4 reference strain	18	6–31

*Abbreviations*: 95% CI, 95% confidence interval; DENV, dengue virus; FRNT, foci reduction neutralization test

Four study participants had similar FRNT_90_ titers against two serotypes (one with DENV-1 + DENV-2, one with DENV-3 + DENV-4, two with DENV-1 + DENV-4). The FRNT_90_ titers for the DENV-1 reference strain (mean FRNT_90_ = 201) of the seropositive sera (*n* = 29) were significantly higher than against DENV-2 (mean FRNT_90_ = 13; Mann-Whitney *U* = 114.5, *n*_1_ = *n*_2_ = 29, *P* < 0.0001, two-tailed), DENV-3 (mean FRNT_90_ = 12; Mann-Whitney *U* = 110, *n*_1_ = *n*_2_ = 29, *P* < 0.0001, two-tailed) and DENV-4 (mean FRNT_90_ = 18; Mann-Whitney *U* = 117, *n*_1_ = *n*_2_ = 29, *P* < 0.0001, two-tailed; Table 3, Fig. 2). The FRNT_90_ titers against the genotype V (mean FRNT_90_ = 444) were twice as high as the titers against the DENV-1 reference strain (mean FRNT_90_ = 201) belonging to genotype I in the positive individuals (*n* = 29). As observed before with the DENV-1 reference strain, the mean FRNT_90_ titer against the DENV-1 genotype V strain was significantly higher than the mean neutralization titers against the other DENV serotypes (DENV-2: Mann-Whitney *U* = 42, *n*_1_ = *n*_2_ = 29, *P* < 0.0001, two-tailed; DENV-3: Mann-Whitney *U* = 39, *n*_1_ = *n*_2_ = 29, *P* < 0.0001, two-tailed; DENV-4: Mann-Whitney *U* = 53.5, *n*_1_ = *n*_2_ = 29, *P* < 0.0001, two-tailed; Table 3, Fig. 2).

None of the 71 tested sera showed any detectable neutralizing antibodies against JEV. The results of the 32 serum samples formerly tested negative in both indirect ELISAs (in-house and Panbio) were confirmed negative by FRNT with all DENV reference strains and the DENV-1 genotype V isolate.

### ELISAs in comparison to FRNT

The second objective of this study was the evaluation of the DENV-1 ED3 dimer indirect ELISA in comparison to the commercial IgG indirect ELISA from Panbio and the FRNT. This comparative analysis was done with a subset of 71 sera tested in all above mentioned assays. Within these, the in-house DENV-1 ED3 dimer indirect ELISA detected 28 (39.4%) positive individuals, whereas the commercial DENV indirect ELISA detected IgG antibodies in 32 of the individuals (45.1%; Table 2). For three samples, the results with the Panbio indirect ELISA were undetermined. The FRNT confirmed 78.6% (22/28) of the positive results from the in-house indirect ELISA results and 90.6% (29/32) of the Panbio indirect ELISA positive results (Additional file 4: Table S2). Taking the FRNT as the gold standard assay, the in-house ELISA produced six false positive and seven false negative results. The Panbio indirect ELISA produced three false positive results. Interestingly, one individual who tested positive with the indirect Panbio ELISA was negative when tested by the in-house ELISA and the FRNT using DENV reference strains, but had detectable neutralizing antibodies against DENV-1 genotype V (FRNT_90_ = 30). Including all 71 samples tested with FRNT, the DENV-1 in-house ELISA led to 58 congruent results (81.7%) with the FRNT (κ = 0.619; 95% CI: 0.433–0.806). The kappa test revealed 91.5% agreement (65/71 results) between the Panbio indirect ELISA and the FRNT (κ = 0.836, 95% CI: 0.716–0.956).

The five individuals identified as having had a secondary DENV infection with the IgG capture ELISA from Panbio showed no significantly higher titers of neutralizing antibodies in the FRNT. However, these individuals with suspected secondary DENV infections had more often a broad neutralization pattern, where the FRNT analysis did not identify a single serotype but detected multiple serotypes. Three of these secondary infected individuals belong to the age group of 60+.

## Discussion

The results of our dengue seroprevalence study on the population of Madeira Island are in agreement with investigations made during or directly after the outbreak. The extent of the outbreak and the fact that Madeira is a favourite tourist destination led to 81 exported cases to mainland Europe after returning from Madeira: 11 cases to mainland Portugal and 70 in other European countries [18] including Finland [32], Belgium [33], Romania [34], Germany and the UK [35]. Partial sequencing and phylogenetic investigations of autochthonous cases from Madeira and imported cases to other European countries identified DENV serotype 1 (DENV-1) genotype V as the causative agent of the outbreak [32, 36], highly likely imported from Venezuela [20, 34].

Our study using FRNT confirmed this suspicion, revealing that the concentration of neutralizing antibodies was higher against genotype V than against the reference isolate of genotype I. DENV-1 genotype V is epidemiologically very important due to its broad distribution in the Americas, Asia, Oceania and Africa over the last 75 years [37, 38], and because it can cause infections with a severe clinical outcome [39].

The distribution of the seropositive individuals found in this study confirmed the higher attack rate in the southern districts, especially in Funchal, as reported after the ECDC outbreak investigation [18]. This is not surprising as the densities of both the human and *Ae. aegypti* populations are highest on the southern coastline. The high seroprevalence in individuals aged 60+ could be due to unreported previous exposure to DENV. This is supported by our findings that three out of the five identified secondary infections occurred in this age group.

We found neutralizing antibodies, mainly directed against DENV-1, in 7.8% of our study population, whereas the infection rate based on the 1080 confirmed cases of the outbreak was only 0.4% (and ~1% for probable cases). A similarly high seroprevalence rate (5%) was observed in individuals without recollection of symptoms in the vicinity of the Tokyo Yoyogi Park outbreak that led to 162 confirmed cases [16]. A 7:1 ratio of subclinical to symptomatic infection outcome is within the range previously observed [3, 40]. Due to the high number of subclinical or inapparent infections, especially in naïve populations, seroprevalence studies are important for risk assessment if DENV-1 re-emerges or if another DENV serotype is imported [3]. From our seroprevalence studies, global estimates of infection in the Madeira population suggest a very high number of individuals have been exposed to the virus during this relatively short epidemic. This is significant as secondary infections lead to an increase in severity of disease [41].

Overall, the cross-reactivity of DENV is not fully understood and there are contrary findings. The specificity of antibodies is not directly correlated with their neutralization capacity. Serotype-specific antibodies represent only a small fraction of the neutralizing antibodies [42] and cross-reacting antibodies contribute significantly to the neutralization capacity [43, 44]. Additionally, neutralizing antibodies are an important correlate for protection [45] but are not the only determining factor. Moreover, recent findings highlight the importance of the cellular immune response for protective immunity [46]. It is assumed that a primary DENV infection induces a short-term cross-reacting immune response against all serotypes [47, 48], but no long-term protection against heterotypic secondary DENV infection [49]. However, there is long-term homotypic humoral immunity (e.g. DENV-1 antibodies were found even 60 years after the infection [50]) although there are recent reports of homotypic DENV re-infections with a clinical outcome [51, 52]. Even cross-protection across genotypes within one serotype varies, as is known for DENV-2 [53, 54] and DENV-3 [55]. On the other hand vaccination studies in macaques showed cross-protection between the two DENV-1 genotypes IV and V [56]. Cross-neutralization among the DENV-1 genotypes was also observed in patients infected with genotype I or IV [57]. However, studies with mouse monoclonal antibodies against DENV-1 genotype II showed a reduced neutralization capacity against heterologous genotypes [58]. Additionally, Shrestha et al. [58] investigated DENV-1 monoclonal antibodies from mice infected with DENV-1 genotype II and found only two out of 76 monoclonal antibodies that showed a strong neutralization against all five DENV-1 genotypes. Such partial lack of intra-serotype cross-neutralization can be explained by the high diversity of the DENV-1 serotype [59] and may explain our observed differences in neutralization to genotypes I and V of DENV-1.

The second goal of this investigation was the evaluation of the previously developed ED3 dimer indirect ELISA [28]. This assay showed a good agreement of 81.7% with the gold standard for flavivirus serological diagnostics, the neutralization test, performed in this study as FRNT with DENV reference strains. The commercial indirect ELISA of Panbio showed 91.5% congruent results with the FRNT using samples from a non-flavivirus endemic area. This study demonstrates the feasibility of seroprevalence analysis with in-house ED3 dimer indirect ELISA. The lower sensitivity compared to the Panbio indirect ELISA and the FRNT could be explained by the use of the DENV-1 ED3 antigen alone and not in combination with the respective antigens of the other serotypes. A combined application of recombinant antigens for all four serotypes could lead to the detection of the individuals with more cross-reactive antibodies, as we observed study participants with FRNT serotypes other than DENV-1 and four individuals with similar titers of neutralizing antibodies against two serotypes. The strategy of combining recombinant antigens of all four DENV serotypes has been already used successfully in diagnostic assays [60–62]. Additionally, as described by Zidane et al. [28], the DENV-1 sequence used for the production of the DENV-1 ED3 antigen is closest to the PCP-consensus sequence derived from 600 DENV strains including all four serotypes [63].

Further surveillance of invasive mosquitoes and vector-borne diseases on Madeira Island is important not only regarding the still existing risk of (re-)emerging DENV, but also due to the high danger of importation of other arboviruses such as chikungunya or Zika. For the latter, a recent investigation by Jupille et al. [64] showed that *Ae. aegypti* from Madeira Island are efficient vectors. The mosquito surveillance in Funchal observed increasing populations from 2006 and 2008 [65] and the highest mosquito density around Funchal during the beginning of the DENV outbreak [66], likely due to mild climatic conditions.

However, our study has some limitations. For further characterization of the immune response we used a DENV-1 genotype V isolated from a patient in French Guiana in 2009. This isolate belongs to the same genotype as the virus that was partially sequenced during the Madeira outbreak. Nevertheless, to our knowledge the Madeira virus was never isolated, which hindered the direct analysis of the immune response against this virus. Another limitation is that not all districts were represented equally. Most of the study participants were residents of Funchal, which might lead to an underrepresentation of cases in the other districts.

## Conclusions

We performed the first seroprevalence study after the DENV-1 outbreak on Madeira Island. We observed a predominant immune response against DENV-1, especially against genotype V, in seropositive study participants. Our study also revealed that the number of infections might have been much higher than estimated from only confirmed cases in 2012/2013, as we observed a seroprevalence of 7.8%. These mainly DENV-1 immune individuals are not protected from a secondary DENV infection and the majority of the population of Madeira Island is still naïve for DENV and other arboviruses. Therefore, the surveillance of mosquitoes and arboviruses should be continued on Madeira Island as well as in other European areas where invasive vector mosquitoes are present.

## Additional files

**Additional file 1.** STROBE Statement. Checklist for reports of observational studies.

**Additional file 2: Table S1.** Expected population sample distribution, by gender and age group.

**Additional file 3: Figure S1.** Phylogenetic tree of complete sequence of the E gene of DENV-1 from Madeira. The complete sequence of the E gene was analysed and assembled by using the CLC Main Workbench 5.5 package (CLC bio A/S, Aarhus, Denmark). MAFFT alignment software was used to perform multiple sequences alignment of Madeira DENV-1 strains with other reference strains from genotypes I, IV and V available in GenBank. Phylogenetic analyses were performed in MEGA7 software using the maximum-likelihood method with a general time reversible model. Bootstrap values, indicated at the nodes, were obtained from 1000 bootstrap replicates and are reported as percentages. DENV-1 French Guiana 2009 strain (genotype V) and DENV-1 Hawaii reference strain (genotype I) used or for the foci reduction neutralization test are indicated by red colour. The DENV-1 strains from Madeira outbreak (genotype V) in 2012 are indicated by blue colour. The ID of each sequence is structured as follows: GenBank accession no_country_name_year of isolation. The scale-bar indicates nucleotide substitutions per site.

**Additional file 4: Table S2.** Serological results of ELISA-positive individuals and comparison with foci reduction neutralization test (FRNT) (*n* = 39).

## Abbreviations

CI: confidence interval
DENV: dengue virus
YFV: yellow fever virus
ELISA: enzyme-linked immunosorbent assay
FRNT: foci reduction neutralization test
IgG: immunoglobulin G
