# Supplementary material for: First dengue virus seroprevalence study on Madeira Island after the 2012 outbreak indicates unreported dengue circulation
Source: Parasit Vectors. 2019 Mar 13;12:103. doi: 10.1186/s13071-019-3357-3 (PMC6417143; doi:10.1186/s13071-019-3357-3)
Supplement: Supplementary file 1 — Additional file 1. STROBE Statement. Checklist for reports of observational studies. [file 13071_2019_3357_MOESM1_ESM.docx]

**Additional file 1:** STROBE Statement - Checklist for reports of observational studies

|  | Item No | Recommendation | Manuscript |
| --- | --- | --- | --- |
| **Title and abstract** | 1 | (*a*) Indicate the study’s design with a commonly used term in the title or the abstract | Abstract, paragraph 2 |
|  |  | (*b*) Provide in the abstract an informative and balanced summary of what was done and what was found | Abstract, paragraph 2 and 3 |
| Introduction | | |  |
| Background/ rationale | 2 | Explain the scientific background and rationale for the investigation being reported | Introduction, paragraph 1 and 2 |
| Objectives | 3 | State specific objectives, including any pre-specified hypotheses | Introduction, paragraph 3 |
| Methods | | |  |
| Study design | 4 | Present key elements of study design early in the paper | Methods, Study design and sampling, paragraph 1 |
| Setting | 5 | Describe the setting, locations, and relevant dates, including periods of recruitment, exposure, follow-up, and data collection | Methods, Study design and sampling, paragraph 1 |
| Participants | 6 | (*a*) Give the eligibility criteria, and the sources and methods of selection of participants | Methods, Study design and sampling, paragraph 2 |
| Variables | 7 | Clearly define all outcomes, exposures, predictors, potential confounders, and effect modifiers. Give diagnostic criteria, if applicable | N/A |
| Data sources/ measurement | 8* | For each variable of interest, give sources of data and details of methods of assessment (measurement). Describe comparability of assessment methods if there is more than one group | N/A |
| Bias | 9 | Describe any efforts to address potential sources of bias | N/A |
| Study size | 10 | Explain how the study size was arrived at | Methods, Study design and sampling, paragraph 1, and Statistical analysis, paragraph 2 |
| Quantitative variables | 11 | Explain how quantitative variables were handled in the analyses. If applicable, describe which groupings were chosen and why | N/A |
| Statistical methods | 12 | (*a*) Describe all statistical methods, including those used to control for confounding | Methods, Statistical analysis, paragraph 1 |
|  |  | (*b*) Describe any methods used to examine subgroups and interactions | Methods, Statistical analysis, paragraph 1 |
|  |  | (*c*) Explain how missing data were addressed | N/A |
|  |  | (*d*) If applicable, describe analytical methods taking account of sampling strategy | N/A |
|  |  | (*e*) Describe any sensitivity analyses | N/A |

|  | Item No | Recommendation | Manuscript |
| --- | --- | --- | --- |
| Results | | |  |
| Participants | 13* | (a) Report numbers of individuals at each stage of study—eg numbers potentially eligible, examined for eligibility, confirmed eligible, included in the study, completing follow-up, and analysed | Results, Study population, paragraph 1 |
|  |  | (b) Give reasons for non-participation at each stage | N/A |
|  |  | (c) Consider use of a flow diagram | N/A |
| Descriptive data | 14* | (a) Give characteristics of study participants (eg demographic, clinical, social) and information on exposures and potential confounders | Results, Study population, paragraph 1, and Table 1 |
|  |  | (b) Indicate number of participants with missing data for each variable of interest | N/A |
| Outcome data | 15* | Report numbers of outcome events or summary measures | Results, Seroprevalence, paragraph 1 |
| Main results | 16 | (*a*) Give unadjusted estimates and, if applicable, confounder-adjusted estimates and their precision (eg, 95% confidence interval). Make clear which confounders were adjusted for and why they were included | N/A |
|  |  | (*b*) Report category boundaries when continuous variables were categorized | N/A |
|  |  | (*c*) If relevant, consider translating estimates of relative risk into absolute risk for a meaningful time period | N/A |
| Other analyses | 17 | Report other analyses done—eg analyses of subgroups and interactions, and sensitivity analyses | N/A |
| Discussion | | |  |
| Key results | 18 | Summarise key results with reference to study objectives | Discussion, paragraph 2 and 3 |
| Limitations | 19 | Discuss limitations of the study, taking into account sources of potential bias or imprecision. Discuss both direction and magnitude of any potential bias | Discussion, paragraph 7 |
| Interpretation | 20 | Give a cautious overall interpretation of results considering objectives, limitations, multiplicity of analyses, results from similar studies, and other relevant evidence | Discussion, paragraph 2 and 3 |
| Generalisability | 21 | Discuss the generalisability (external validity) of the study results | N/A |
| Other information | | |  |
| Funding | 22 | Give the source of funding and the role of the funders for the present study and, if applicable, for the original study on which the present article is based | Acknowledgements, paragraph 1 |

*Give information separately for exposed and unexposed groups.
