# Supplementary material for: First dengue virus seroprevalence study on Madeira Island after the 2012 outbreak indicates unreported dengue circulation
Source: Parasit Vectors. 2019 Mar 13;12:103. doi: 10.1186/s13071-019-3357-3 (PMC6417143; doi:10.1186/s13071-019-3357-3)
Supplement: Supplementary file 2 — Additional file 2: Table S1. Expected population sample distribution, by gender and age group. [file 13071_2019_3357_MOESM2_ESM.docx]

# Additional file 2: Table S1. Expected population sample distribution, by gender and age group.

| Age group | Male | Female | Total |
| --- | --- | --- | --- |
| 10-19 years | 17 | 15 | 32 |
| 20-29 years | 17 | 17 | 34 |
| 30-39 years | 19 | 20 | 39 |
| 40-49 years | 20 | 21 | 41 |
| 50-59 years | 17 | 20 | 37 |
| 60+ years | 21 | 33 | 54 |
| Total | **111** | **126** | **237** |
