# Supplementary material for: First dengue virus seroprevalence study on Madeira Island after the 2012 outbreak indicates unreported dengue circulation
Source: Parasit Vectors. 2019 Mar 13;12:103. doi: 10.1186/s13071-019-3357-3 (PMC6417143; doi:10.1186/s13071-019-3357-3)
Supplement: Supplementary file 4 — Additional file 4: Table S2. Serological results of ELISA-positive individuals and comparison with foci reduction neutralization test (FRNT) (n = 39). [file 13071_2019_3357_MOESM4_ESM.docx]

# Additional file 4: Table S2. Serological results of ELISA-positive individuals and comparison with foci reduction neutralization test (FRNT) (n=39).

|  | FRNT_90_ | | | | | | |  | ELISAs | | |
| --- | --- | --- | --- | --- | --- | --- | --- | --- | --- | --- | --- |
| Serum no. | **DENV-1*** | **DENV-1** | **DENV-2** | **DENV-3** | **DENV-4** | **JEV** | **DENV Serotype** |  | **ED3 dimer (in-house)** | **Panbio IgG indirect ELISA** | **Panbio IgG capture ELISA** |
| 3 | **716** | **348** | 0 | 0 | 0 | 0 | DENV-1 |  | **Pos** | **Pos** | Neg |
| 10 | **362** | **177** | 0 | 0 | 0 | 0 | DENV-1 |  | **Pos** | **Pos** | **Pos** |
| 20 | 0 | 0 | **51** | 0 | 0 | 0 | DENV-2 |  | Neg | **Pos** | Neg |
| 22 | **134** | **56** | 0 | 0 | 0 | 0 | DENV-1 |  | Neg | **Pos** | Neg |
| 27 | 0 | 0 | 0 | 0 | 0 | 0 | - |  | **Pos** | Neg | Neg |
| 45 | 0 | 0 | 0 | 0 | 0 | 0 | - |  | **Pos** | Neg | Neg |
| 47 | **169** | **125** | **104** | 0 | **20** | 0 | DENV-1, -2 |  | Neg | **Pos** | **Pos** |
| 48 | **62** | **22** | 0 | 0 | 0 | 0 | DENV-1 |  | **Pos** | **Pos** | Neg |
| 69 | **73** | 0 | 0 | **58** | 0 | 0 | DENV-1*, -3 |  | **Pos** | **Pos** | *Undetermined* |
| 76 | **826** | **261** | **30** | **72** | **26** | 0 | DENV-1 |  | **Pos** | **Pos** | **Pos** |
| 84 | **1774** | **320** | 0 | 0 | 0 | 0 | DENV-1 |  | **Pos** | **Pos** | Neg |
| 96 | **740** | **199** | 0 | 0 | 0 | 0 | DENV-1 |  | **Pos** | **Pos** | Neg |
| 103 | 0 | 0 | 0 | 0 | 0 | 0 | - |  | **Pos** | **Pos** | Neg |
| 108 | **113** | **134** | 0 | 0 | **90** | 0 | DENV-1, -4 |  | **Pos** | **Pos** | **Pos** |
| 111 | **251** | **342** | 0 | 0 | 0 | 0 | DENV-1 |  | **Pos** | **Pos** | *Undetermined* |
| 121 | **30** | **29** | 0 | 0 | 0 | 0 | DENV-1 |  | **Pos** | **Pos** | Neg |
| 129 | **189** | **109** | 0 | 0 | 0 | 0 | DENV-1 |  | **Pos** | **Pos** | Neg |
| 138 | **209** | **179** | 0 | 0 | 0 | 0 | DENV-1 |  | **Pos** | **Pos** | Neg |
| 141 | **173** | **201** | 0 | 0 | 0 | 0 | DENV-1 |  | **Pos** | **Pos** | Neg |
| 143 | 0 | 0 | 0 | 0 | 0 | 0 | - |  | **Pos** | Neg | Neg |
| 144 | 0 | 0 | 0 | 0 | 0 | 0 | - |  | Neg | *Undetermined* | Neg |
| 147 | **604** | **648** | 0 | **20** | 0 | 0 | DENV-1 |  | **Pos** | **Pos** | Neg |
|  | **FRNT_90_** | | | | | | |  | **ELISAs** | | |
| Serum no. | **DENV-1*** | **DENV-1** | **DENV-2** | **DENV-3** | **DENV-4** | **JEV** | **DENV serotype** |  | **ED3 dimer (in-house)** | **Panbio IgG indirect ELISA** | **Panbio IgG capture ELISA** |
| 153 | **71** | 0 | 0 | **24** | **81** | 0 | DENV-1*, -3,-4 |  | **Pos** | **Pos** | Neg |
| 165 | **320** | **148** | 0 | 0 | 0 | 0 | DENV-1 |  | **Pos** | **Pos** | *Undetermined* |
| 183 | **1063** | **777** | 0 | 0 | 0 | 0 | DENV-1 |  | **Pos** | **Pos** | Neg |
| 190 | **1606** | **402** | **21** | **32** | **44** | 0 | DENV-1 |  | **Pos** | **Pos** | **Pos** |
| 199 | 0 | 0 | 0 | 0 | 0 | 0 | - |  | **Pos** | *Undetermined* | Neg |
| 201 | 0 | 0 | 0 | 0 | **141** | 0 | DENV-4 |  | Neg | **Pos** | Neg |
| 210 | **21** | **20** | 0 | 0 | **35** | 0 | DENV-1, -4 |  | **Pos** | **Pos** | Neg |
| 224 | 0 | 0 | 0 | 0 | 0 | 0 | - |  | Neg | **Pos** | Neg |
| 231 | 0 | 0 | 0 | 0 | 0 | 0 | - |  | **Pos** | Neg | Neg |
| 265 | **835** | **401** | 0 | 0 | 0 | 0 | DENV-1 |  | **Pos** | **Pos** | Neg |
| 283 | **30** | 0 | 0 | 0 | 0 | 0 | DENV-1* |  | Neg | **Pos** | Neg |
| 291 | 0 | 0 | 0 | 0 | 0 | 0 | - |  | Neg | *Undetermined* | Neg |
| 293 | **715** | **338** | 0 | 0 | 0 | 0 | DENV-1 |  | **Pos** | **Pos** | Neg |
| 306 | **355** | **145** | 0 | 0 | 0 | 0 | DENV-1 |  | Neg | **Pos** | Neg |
| 309 | 0 | 0 | 0 | 0 | 0 | 0 | - |  | Neg | **Pos** | Neg |
| 326 | **278** | **79** | 0 | 0 | 0 | 0 | DENV-1 |  | Neg | **Pos** | Neg |
| 373 | **1133** | **344** | **23** | 0 | 0 | 0 | DENV-1 |  | **Pos** | **Pos** | *Undetermined* |

ELISA: enzyme-linked immuno-sorbent assay; DENV: dengue virus; DENV-1: dengue virus serotype 1; DENV-2: dengue virus serotype 2; DENV-3: dengue virus serotype 3; DENV-4: dengue virus serotype 4; *DENV-1 genotype V; Pos: positive; Neg: negative; **bold**: positive test results; underlined: serotype identified as DENV with the highest FRNT_90_ titer (at least 4-fold higher than second highest FRNT_90_ titer).
